# The Role of Stress and Mineralocorticoid Receptor Haplotypes in the Development of Symptoms of Depression and Anxiety During Adolescence

**DOI:** 10.3389/fpsyt.2020.00367

**Published:** 2020-05-15

**Authors:** Hinke M. Endedijk, Stefanie A. Nelemans, Remmelt R. Schür, Marco P. M. Boks, Pol van Lier, Wim Meeus, Susan Branje, Christiaan H. Vinkers

**Affiliations:** ^1^Department of Youth and Family, Utrecht University, Utrecht, Netherlands; ^2^Department of Psychiatry, University Medical Center Utrecht, Utrecht, Netherlands; ^3^Clinical Developmental Psychology, Vrije Universiteit Amsterdam, Amsterdam, Netherlands; ^4^Department of Psychiatry, Amsterdam University Medical Center, Amsterdam, Netherlands; ^5^Department of Anatomy and Neurosciences, Amsterdam University Medical Center, Amsterdam, Netherlands

**Keywords:** depression, anxiety, mineralocorticoid receptor (MR), stress, adolescence, parenting, development

## Abstract

Adolescence is a critical developmental period characterized by heightened levels of depressive and anxiety symptoms. Experiencing chronic or environmental stress, for example, as a result of traumatic events or insensitive parenting, increases the risk for depression and anxiety. However, not all adolescents develop depressive or anxiety symptoms following environmental stressors, due to differences in stress resilience. One of the factors involved in stress resilience is enhanced functionality of the mineralocorticoid receptor (MR), one of the two brain receptors for the stress hormone cortisol. High levels of MR functionality result in relatively lower rates of depression, particularly in women that experienced stress. However, much less is known about MR functionality in relation to the development of adolescent depression and to other internalizing behavior problems such as anxiety. We therefore examined whether the effects of a functional MR haplotype (i.e., the MR CA haplotype) on the development of depressive and anxiety symptoms are sex-dependent, as well as interact with environmental stressors. In a community sample of adolescents (*N* = 343, 9 waves between age 13 and 24), environmental stressors were operationalized as parental psychological control and childhood trauma. Results showed a sex-dependent effect of MR CA haplotype on the development of depressive symptoms but not for anxiety symptoms. MR CA haplotypes were protective for girls but not for boys. This study sheds more light on the sex-dependent effects of MR functionality related to the development of depressive and anxiety symptoms during adolescence.

## Introduction

Adolescence is a vulnerable period for the development of internalizing behavior problems. While rates of depressive and anxiety symptoms are generally low in childhood, they increase to near-adult prevalence levels in adolescence (1, 2), with some studies showing a six-fold increase in rates of depression from age 15 to age 18 (1). Moreover, girls experience depressive and anxiety symptoms twice as often as boys (1, 3). High levels of stressors are a risk factor for the development of internalizing behavior problems (1, 4–6). In addition to the experience of stressful events, individual differences in stress resilience are consistently related to risk for psychiatric disorders (7). There is increasing evidence that the mineralocorticoid receptor (MR), one of the two receptors for the stress hormone cortisol, is important for stress resilience (8, 9). Functional MR receptor haplotypes (typical combinations of genetic variants with consequences for MR expression and activity), have been repeatedly found to affect the link between stressors and internalizing disorders (10, 11). Specifically, MR effects appear to be sex-dependent, with a stronger protective effect of increased constitutional MR activity for females compared to males (10, 11). However, much less is known about the relation of functional MR receptor haplotypes, environmental stressors, and sex with the development of depressive and anxiety symptoms during adolescence. We therefore investigated the possible sex-specific associations of the common and functional MR haplotype— both in presence and absence of environmental stressors—on depressive and anxiety symptoms during the crucial developmental period of adolescence.

In order to understand the consequences of MR haplotype and environmental stressors on the development of internalizing behavior problems such as depressive and anxiety symptoms, we focused on two common and salient environmental stressors during childhood or adolescence: childhood trauma in the form of physical abuse or neglect, and parental psychological control, which can be considered a form of emotional abuse. Childhood trauma and psychological control can have a long-lasting impact, which may become visible particularly during adolescence (6). When parents are psychologically controlling, they display disappointment and children feel pressured and guilty that they did not comply with the parent’s requests, or anxious about losing the parent’s approval (12). Also, childhood abuse is characterized by guilt and self-blame of the child (13). These processes may both result in loss of confidence and excessive inappropriate guilt, symptoms often present in internalizing disorders like depression (4, 14) and anxiety (3).

The MR is important for a well-functioning HPA axis. In response to stress, the HPA axis is rapidly activated and results in the release of cortisol (7). Cortisol binds to MRs in the brain and is essential for the activation and restoration of HPA axis activity in relation to stress (8, 15). Upon binding of cortisol, MRs translocate into the nucleus where they act as transcription factor by binding to responsive elements in promotor regions of target genes to increase gene expression (16, 17). Several clinical studies have pointed to a role of the MR in relation to the consequences of stress for depression. In rodents, decreased MR expression was associated with higher levels of depressive-like behavior (18) and rodents with elevated levels of MR functioning showed less anxious behaviors (19–22). In humans, MR studies with respect to internalizing behavior problems are scarce, and exclusively focus on depression. Several studies both in clinical and population-based adult samples have shown decreased MR expression in patients with Major Depressive Disorder (10, 23, 24). Also, lower rates of depression were found in human adults with higher MR expression (25).

MR is encoded by the NR3C2 gene, located on chromosome 4 (26). A sequencing study in a Dutch cohort identified two single-nucleotide polymorphisms (SNPs) in this gene, MR-2C/G (rs2070951) and MRI180V (rs5522), that affect the transactivational capacity in response to stress hormones (27). These SNPs constitute four possible haplotypes; CA, CG, GA, and GG, although the latter is very rare (28, 29). Mainly, the CA haplotype seems to be related to stress resilience. Having more CA haplotypes could therefore be protective against the negative consequences of environmental stressors and therefore the development of depressive and anxiety symptoms during adolescence. In this study, we will explicitly focus on the MR CA haplotype.

The MR CA haplotype has been shown to have sex-dependent effects on depression (10, 11). These sex differences may be explained by the fact that the female hormones estrogen and progesterone bind to the MR and affect MR functionality, although the exact underlying mechanism is unclear (30). Sex-specific effects of the MR may even be pronounced during adolescence when levels of female hormones increase and influence the maturing HPA axis (31).

In the current study, we therefore examined whether there is a sex-specific role of functional genetic variation in the MR (CA haplotype) for the development of depressive and anxiety symptoms during adolescence and young adulthood, and whether putative sex-specific MR effects depend on environmental stressors. We aimed to extend previous findings on sex-dependent MR effects on adult depression to the crucial developmental period of adolescence, and to extend the focus of MR on depression to anxiety as another important internalizing behavior problem. We hypothesized that the functional and common MR CA haplotype would have sex-dependent effects on the development of depressive and anxiety symptoms, and that the CA haplotype would be protective in girls. Moreover, we hypothesized that these protective effects of the MR CA haplotype in girls would be the most outspoken after exposure to environmental stressors.

## Methods

### Participants

Participants were 343 Dutch adolescents (55.7% boys) with a mean age of 13 years (SD = .4) and all attending the first grade of secondary school at the first wave of data collection in 2005. Participants took part in the longitudinal population-based RADAR-Young study (Research on Adolescent Development And Relationships), in which 522 adolescents originally participated. Adolescents were recruited in the center and west of Netherlands. The first six waves were annual until the age of 18 and the last 3 waves were biennial until the age of 24. All 343 adolescents that still participated at the age of 23 and for whom we collected valid and qualitatively good genetic data were included as participants of the current study. Adolescents from the genetic sample were not different from adolescents from the total sample on sex, age during the first wave, parental psychological control, childhood trauma, and depressive or anxiety symptoms (all *p*s < .05). For the participants in the genetic sample, there were few missing data, with 72% of the adolescents participating in all waves. Moreover, the pattern of missing values was estimated by Little’s (32) Missing Completely at Random test. Although this test was significant (χ2(N = 343, df = 709) = 844.39, *p* < 0.001), the χ^2^/df ratio of 1.19 indicated a good fit between the sample scores with and without imputation (33). Therefore, Full Information Maximum Likelihood was used to handle missing data in the growth curve analyses in M*plus* version 8.0. The study has been approved by the board of the local research institute and by the Medical Ethical Committees of the Utrecht Medical Centre and VU University Medical Centre, Netherlands, and written informed consent was obtained from both adolescents and parents.

### Measures

#### MR CA Haplotypes

Genotyping of all participants was performed using Affymetrix 6.0 array’s (34) on DNA from saliva. Two commonly investigated functional SNPs in the gene encoding the MR (rs2070951 and rs5522) were selected to derive MR haplotypes (15, 28). Rs5522 was genotyped and rs2070951 was imputed from the array data as previously reported (35). In short, all SNPs were strand aligned to the 1,000 genomes Phase 3 release, phased using SHAPEIT V2.970 and imputed to the 1,000 genomes reference with IMPUTE 2.3.1 following standard protocols. SNPs with R^2^ values > .08 and average call rates > 0.99 were retained. Subsequently, SNPHAP was used to derive the haplotypes. This resulted in the following distribution of CA haplotypes: 41.40% adolescents with zero CA haplotypes, 46.36% with one CA haplotype, and 12.24% with two CA haplotypes, with more CA haplotypes indicating higher MR functioning, which is consistent with previous literature (11, 28, 29). Therefore, in the current study, we used the number of MR CA haplotypes as a continuous predictor. There were no significant differences in the haplotype distribution between men and women (*X*^2^ = 2.58, *p* = .276).

#### Environmental Stressors

##### Parental Psychological Control

Psychological control was one of the environmental stressors. Psychological control involves attempts that intrude or manipulate the thinking processes, self-expression, and emotions of the child (36) and can therefore be considered as emotional abuse. It was measured by the Dutch version of the Psychological Control Scale (36), which consists of eight 5-point items on a 5-point Likert scale ranging from not at all applicable to completely applicable. An example item is “My mother acts like she knows what I’m thinking or feeling”. Adolescents reported on psychological control by mother and father separately. All questions were completed from Wave 1 until Wave 7. At the last two waves, these questionnaires were not administered as many adolescents moved out of the parental home during the final waves. The internal consistency was high across waves, ranging from .75 to .89 for the reports about father and from .83 to .88 for the reports about mother. Power was too low to take parental psychological control into account as time-varying covariate. Therefore, for each adolescent, a mean score across the several waves and across fathers and mothers was calculated and standardized. Correlations between waves ranged from .37 to .76 for fathers, from .25 to .67 for mothers, and correlations between mothers and fathers on the same wave ranged from .56 to .72.

##### Childhood Trauma

To measure adolescents’ traumatic events over the whole course of childhood and adolescence, the Dutch version of the Childhood Trauma Questionnaire-Short Form was assessed (37). This questionnaire was administered in the community sample at the ninth wave of data collection (around age 24). This retrospective questionnaire includes four 5-item subscales: physical abuse, emotional abuse, physical neglect, and emotional neglect, and one 4-item subscale: sexual abuse, reflecting the frequency of maltreatment on a 5-point Likert scale ranging from never true to very often true. A fifth item of the sexual abused scale “I believe I was molested” was not included in the Dutch version as there is no proper translation for the word “molested” with a sexual connotation (37). A total standardized continues score was calculated, and the internal consistency of the scale was adequate with a Cronbach’s alpha of .83.

#### Depression

Depressive symptoms were measured by the Dutch adjusted version of the Reynolds Adolescent Depression Scale – 2nd edition (38). This scale consists of 23 items on a 4-point Likert scale ranging from 0 (almost never) to 3 (most of the time) on the subscales dysphoric mood (eight items), negative self-evaluation (eight items), and somatic complaints (seven items). An example item is “I feel bored”. Items were averaged to compute a mean depression score. The internal consistency was high ranging from .93 to .95 on the different waves.

#### Anxiety

Anxiety symptoms were measured by the Screen for Child Anxiety Related Emotional Disorders (39). This scale consists of 38 items on a 3-point Likert scale ranging from 0 (almost never) to 2 (often), and consist of questions about somatic/panic, school phobia, social anxiety, generalized anxiety, and separation anxiety. An example item is “When I feel frightened, it is hard to breathe”. Items were averaged to compute a mean anxiety score. The internal consistency of this scale was high ranging from .90 to .94 on the different waves.

### Analyses

Growth curve analysis in M*plus* version 8.0 was used. To facilitate the interpretation of the effect of the predictor on the developmental trajectory of depressive and anxiety symptoms, a piecewise model was estimated to be able to examine the role of the predictors in different developmental periods. A separate slope was modeled for three developmental periods, resulting in three slopes: one for early adolescence (13–16 years), one for late adolescence (16–20 years), and one for young adulthood (20–24 years). Until age 18, an equidistant time difference of 1 was used for the annual measurements, and after age 18, an equidistant time difference of 2 was used for the biannual measurements. The intercept was the estimated amount of depressive or anxiety symptoms at age 13. The fit of this model was evaluated based on the following criteria: acceptable fit when CFI > .90, RMSEA and SRMR < .10, good fit when CFI > .95, RMSEA < .06 and SRMR < .08 (40).

We used the four latent factors (intercept and three slopes) of the piecewise model as dependent variables to examine the association between stressor, MR (0, 1, or 2 CA haplotypes), sex, and all two-way and three-way interactions with depressive symptoms and anxiety symptoms over the course of adolescence. Four different models were estimated, with either parental psychological control or childhood trauma as environmental stressor, and with either the latent factors of the growth curve of depressive symptoms or the latent factors of the growth curve of anxiety symptoms. This resulted in the following models: a) parental psychological control and depression, b) childhood trauma and depression, c) parental psychological control, and anxiety, d) childhood trauma and anxiety.

## Results

### Descriptives and Model Fit

#### Depression

The piecewise growth model of depressive symptoms yielded a good fit (RMSEA = .062 [CI .043–.081], CFI = .967, SRMR = .032). In general, adolescents showed stable depressive symptoms between age 13 and 16 (β_slope13-16_ = −.126, *p* = .089), after which their symptoms increased between age 16 and 20 (β_slope16–20_ = .328, *p* < .001) and slowly increased between age 20 and 24 (β_slope20–24_ = .144, *p* = .041), but with large individual differences in all parameters (all variances *p*’s < .001). Also, the fit of the growth models with predictors was good, for both the model with parental psychological control (RMSEA = .048 [.033–.062], CFI = .970, SRMR = .023) and the model with childhood trauma (RMSEA = .040 [.025–.053], CFI = .973, SRMR = .029).

#### Anxiety

The piecewise growth model of anxiety symptoms yielded also a good fit (RMSEA = .043 [CI .019–.064], CFI = .982, SRMR = .058). In general, adolescents showed an initial decrease in anxiety symptoms between age 13 and 16 (β_slope13–16_ = −.249, *p* = .006), after which their symptoms increased between age 16 and 20 (β_slope16-20_ = .226, *p* = .005) and stayed stable between age 20 and 24 (β_slope20–24_ = −.102, *p* = .154), but with large individual differences in all parameters (all variances *p*’s < .001). Also, the fit of the growth models with predictors was good, for both the model with parental psychological control (RMSEA = .031 [.009–.047], CFI = .986, SRMR = .038) and the model with childhood trauma (RMSEA = .034 [.017–.048], CFI = .979, SRMR = .047).

### MR, Stressor, and Internalizing Behavior Problems

#### Depression

We found no support for our hypothesis that the MR CA haplotype moderated the effects of environmental stressors on development of depressive symptoms. Specifically, there were no statistically significant three-way interactions between MR CA haplotype, stressors, and sex (all *p*’s > .49, see **Table 1**), indicating no support for sex specific effects of MR in moderating the effect of environmental stressors. Similarly, in absence of sex, the interaction terms of MR CA haplotype with psychological control and MR CA haplotype with childhood trauma were both not significant for the intercept or slopes (all *p*’s > .486).

**Table 1:** Standardized regression coefficients and standard errors of MR, sex, parental psychological control (PCS), and childhood trauma (CTQ) as predictors of the development of depressive symptoms.

	Intercept		Slope 13–16		Slope 16–20		Slope 20–24	
	β	SE	β	SE	β	SE	B	SE
*Parental Psychological control*								
MR	.12	.07	−.13	.08	.04	.09	.00	.10
Sex	.36***	.09	.10	.11	−.17t	.09	.06	.10
PCS	.24*	.10	.18	.12	.02	.12	−.17	.14
MR*sex	−.20*	.09	.11	.12	−.10	.11	−.02	.11
MR*PCS	.02	.10	−.06	.13	−.04	.12	.09	.13
PCS*sex	.19	.13	.12	.15	−.29*	.12	−.02	.14
MR*sex*PCS	.08***	.11	−.02t	.13	.07*	.12	−.05	.13
***R^2^***	.31***		***.10t***		.12*		***.03 n.s.***	
*Childhood trauma*								
MR	.13t	.07	−.10	.09	.04	.09	.00	.09
Sex	.41***	.09	.14	.11	−.19*	.10	.04	.09
CT	.02	.09	.31**	.11	.10	.12	−.11	.11
MR*sex	−.22*	.10	.08	.12	−.08	.11	−.02	.11
MR*CT	.18t	.10	−.02	.11	−.10	.18	.16	.15
CT*sex	.31*	.14	−.12	.22	.00	.14	.12	.14
MR*sex*CT	−.11**	.13	.04*	.19	−.14t	.18	−.17	.17
***R^2^***	.22**		.11*		***.09t***		***.01 n.s.***	

t < .10; *< .05; **< .01; ***< .001; ns, not significant. Sex was coded as boys = 0, girls = 1.

**Figure 1:**
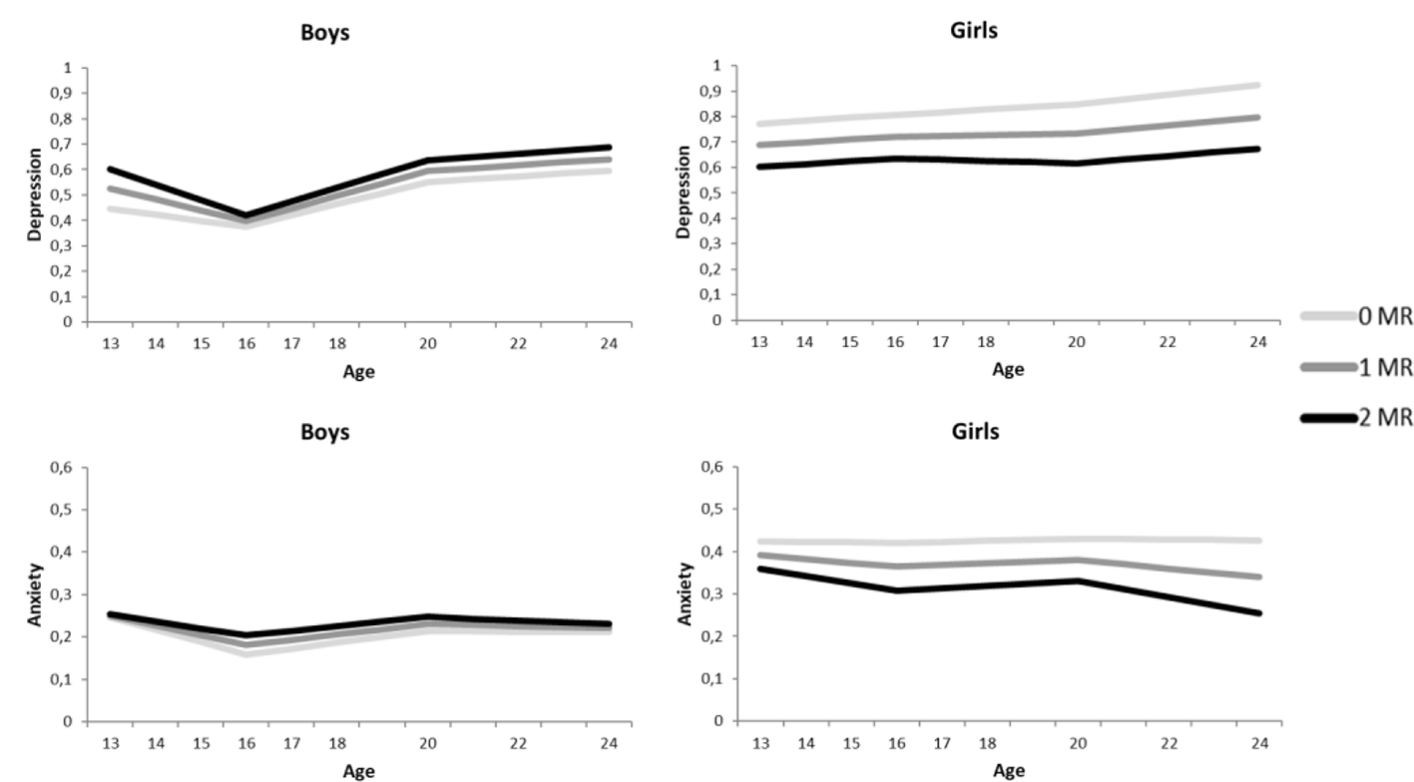
Growth curve models for the depressive symptoms (above) and anxiety symptoms (below), separately for boys (left) and girls (right). The darker the lines the more MR CA haplotypes.

However, in accordance to our hypothesis, we found significant negative interactions between MR CA haplotype and sex on the intercept (β_intercept_ = −.20, *p* = .033 for psychological control, and β_intercept_ = −.22, *p* = .032 for childhood trauma), but not the slopes (all *p*’s > .340). This indicates that girls with more MR CA haplotypes reported lower levels of depressive symptoms across adolescence and young adulthood, while the opposite pattern was found for boys (see **Figure 1**).

Moreover, interaction between environmental stressors and sex were found. On the slope between age 16 and 20, there was a significant interaction effect between psychological control and sex (β_slope16-20_ = −.29, *p* = .021), indicating that girls with higher levels of psychological control increased less in depressive symptoms as compared to girls with lower levels of psychological control, while depressive symptom development for boys was unaffected by parental psychological control. Second, the model with childhood trauma as stress factor showed a significant interaction effect between sex and childhood trauma on the intercept (β_intercept_ = .31, *p* = .028) but not on the slopes (all *p*’s > .394), indicating that girls with higher levels of childhood trauma reported higher levels of depressive symptoms across development.

Above these interaction effects, there were some main effects. Most consistently, sex was found to predict levels of depressive symptoms, with girls showing more depressive symptoms at age 13 (β_intercept_ = .36, *p* < .001 for the parental psychological control model, β_intercept_ = .41, *p* < .001 for the childhood trauma model). In the childhood trauma model, an effect of sex on the second slope (β_slope16-20_ = −.19, *p* = .048) indicated that after an initial higher level of depressive symptoms for girls, between the age of 16 and 20 depressive symptoms in girls increased to a lesser extent compared to boys (potentially due to higher initial or overall levels of depressive symptoms in girls). This effect did not reach significance in the model for parental psychological control (β_slope16-20_ = −.17, *p* = .077), although effect sizes were comparable to the model with childhood trauma. Also, while psychological control was significantly related to more depressive symptoms across development (β_intercept_ = .24, *p* = .019, slopes all *p*’s > .135), childhood trauma did predict a steeper slope in depressive symptoms from age 13 to 16 (β_slope13-16_ = .31, *p* = .005).

#### Anxiety

In accordance with the models of depression, the anxiety models showed no indication of an interaction between MR CA haplotype and environmental stressors, or three-way interactions between MR CA haplotype, sex and environmental stressors on the development of anxiety symptoms (all *p*’s > .233, see **Table 2**). In contrast to depression, no interaction between MR CA haplotype and sex was found on the intercept or the three slopes (all *p*’s > .395). Although **Figure 1** suggests an interaction between MR CA haplotype and sex, the effects are too small to reach significance.

**Table 2:** Standardized regression coefficients and standard errors of MR, sex, parental psychological control (PCS), and childhood trauma (CTQ) as predictors of the development of anxiety symptoms.

	Intercept		Slope 13–16		Slope 16–20		Slope 20–24	
	β	SE	β	SE	β	SE	β	SE
*Parental Psychological control*								
MR	.01	.07	.04	.08	−.02	.08	−.03	.07
Sex	.35***	.09	.20t	.11	−.12	.12	.02	.11
PCS	.21t	.11	−.02	.11	−.07	.11	−.19	.13
MR*sex	−.07	.11	−.11	.14	.03	.13	−.10	.12
MR*PCS	−.09	.11	.07	.12	.00	.11	.13	.11
PCS*sex	.19	.15	.21	.14	−.22	.16	−.03	.15
MR*sex*PCS	.07***	.13	−.12	.16	.10	.15	−.05	.13
***R^2^***	.25***		***.05 n.s.***		***.06 n.s.***		***.04 n.s.***	
*Childhood trauma*								
MR	.01	.07	.06	.08	−.02	.08	−.03	.07
Sex	.40***	.09	.21t	.11	−.14	.11	−.01	.11
CTQ	.04	.09	.07	.10	.06	.13	−.17	.11
MR*sex	−.09	.11	−.11	.13	.04	.13	−.08	.11
MR*CTQ	−.05	.10	.10	.10	−.07	.14	.16	.10
CTQ*sex	.36*	.14	−.06	.16	−.07	.23	.06	.16
MR*sex*CTQ	−.07**	.14	−.06	.17	.06	.24	−.19	.17
***R^2^***	.23**		***.03 n.s.***		***.02 n.s.***		***.04 n.s.***	

t < .10; *< .05; **< .01; ***< .001; ns, not significant. Sex was coded as boys = 0, girls = 1.

Comparable to the models for depression, there was an interaction effect between childhood trauma and sex (β_intercept_ = .36, *p* = .010), but not between parental psychological control and sex (β_intercept_ = .19, *p* = .197), on the intercept of anxiety symptoms. These findings indicate that girls with higher levels of childhood traumas reported higher levels of anxiety symptoms across development.

There were a couple of main effects, for which all effect sizes were comparable to the models for depression. Both anxiety models showed a main effect of sex on the intercept (β_intercept_ = .35, *p* < .001 for parental psychological control, and β_intercept_ = .40, *p* < .001 for childhood trauma), indicating that girls reported higher levels of anxiety as compared to boys across development. Moreover, parental psychological control showed a marginally significant effect on the intercept of anxiety symptoms (β_intercept_ = .21, *p* = .056), with an effect size comparable to depression, suggesting that adolescents that experience higher levels of parental psychological control also reported higher levels of anxiety. In contrast to the depression model, there was no indication of a main effect of childhood trauma on the intercept or the three slopes of anxiety (all *p*’s > .102).

## Discussion

In this longitudinal study of a community sample assessed during adolescence and young adulthood, we found that common and functional MR haplotypes had sex-dependent protective effects on depressive symptoms but not on anxiety symptoms. Specifically, we found that girls with the MR CA haplotype consistently had lower depressive symptoms compared to non-CA haplotype carriers. The MR CA haplotype was a *protective* factor for mean levels of depressive symptoms of girls across adolescence and young adulthood, but a *risk* factor for boys, independent of the level of environmental stressors. Findings support earlier evidence for sex-dependent effects of the functional and common MR CA haplotype on depression. With regard to environmental stressors, we found no support for a moderating role of MR CA haplotype in the effects of environmental stressors on depressive and anxiety symptoms during adolescence, and no sex-specific effects of environmental stressors were found in relation to MR CA haplotype. This study adds to previous findings by shedding more light on the sex-dependent effects of the MR for the development of internalizing behavior problems in relation to stressors during the turbulent period of adolescence.

There were no significant interaction effects between stressors and MR CA haplotype, suggesting that MR CA haplotype is equally protective in low and high stressful environments. However, a study in the same sample showed a positive effect of MR CA haplotype on prosocial behavior, empathic concern and perspective taking for adolescents who experienced high levels of stressors, and negative effects for adolescents who reported low levels of stressors (41). Such a protective effect of MR CA haplotype under circumstances of high stress is in line with the study of Vinkers et al. (11) that found stronger MR CA haplotype effects for higher levels of childhood trauma. This suggests that the MR receptors will mainly be occupied and provide feedback to the HPA axis when stress hormone levels are high and therefore are mainly relevant under circumstances of high stress (8, 15). As such, MRs would be mainly protective in adolescents with high levels of stressors by affecting the appraisal of and response to a stressful situation (9).

One possible explanation for why our study did not find a significant interaction between MR CA haplotype and stressors could be due to the way stressors were measured. First, both questionnaires assessing parental psychological control and childhood traumas assessed the amount of stressors, and not the amount of stress it created for the adolescent. When someone experiences more stressful events, or a longer period of stress, their stressful event load accumulates during lifetime (42), while at the same time the effects of stressful experiences decrease over time (42–44). Therefore, a certain stressor during childhood or adolescent might result in different levels of stress at different points in development. Moreover, by taking one score for the whole period of adolescence and adulthood we lost information about the timing of the stressors. Second, the level of stressors in our community sample was relatively low, which made it less likely to detect effects. Third, both questionnaires mainly focused on parental maltreatment, while other forms of stressful events that might have contributed to adolescents’ stress load and the development of internalizing behavior problems were not taken into account, like peer-victimization (45). Future studies could provide more insight in this process by measuring a broader range of stressful events, asking participants to report the level of stress associated with childhood trauma or parental psychological control, and asking the exact timing and duration of these stressful experiences.

The protective role of MR CA haplotype in girls is in line with earlier studies that found sex-specific effects of MR CA haplotype related to the development of depression in a population sample (10, 11). In line with our results, they found that females with MR CA haplotype were protected against the development of depression, and that males with MR CA haplotype were at increased risk for depression. This sex-specific MR effect may be understood from the fact that the female hormones progesterone and estrogen influence MR functioning (30). This makes females differentially susceptible to the consequences of stress and therefore for the development of depression and other internalizing behavior problems. For example, females with the MR CA haplotypes were found to be less sensitive to their female hormonal status with respect to emotion recognition (46). In line with this finding, females with MR CA haplotypes were protected against the negative effects of oral contraceptives on recognition of sad and fearful faces and worse emotional memory (47). These findings suggest that sex interacts with MR in such way that MR CA haplotype is more protective in women, but that the sex-specific MR effects might depend on female hormone levels. To gain better understanding of the exact sex-specific MR effects on depressive and anxiety symptoms, future studies should take into account the actual hormone levels or focus on the underlying biological mechanism for the sex-specific MR effects.

For girls, MR CA haplotype had a constant significant protective effect on the level of depressive symptoms across the whole period of adolescence and young adulthood, rather than being related to change in depressive symptoms across time. It might be that MR CA haplotype already has an effect on depressive symptom development before the start of adolescence. As MRs are important for an optimal stress response (8, 15), the functional and common MR CA haplotype may already affect stress resilience from birth on, and consequently, the development of the earliest symptoms of depression or other internalizing behavior problems. For example, coping style and temperament are found to interact with environmental stressors and moderate the risk of depression (42). It might be that stress resilience due to a MR CA haplotype is related to a more adaptive coping style or certain temperamental inclinations in young children, and therefore results in lower rates of depression. Childhood studies about the role of MR in internalizing behavior problems could provide more insight in the putatively protective role of MR CA haplotype in the earliest symptoms of depression in relation to hormonal status in males and females.

Although sex-specific MR effects were found for development of depressive symptoms, there was no sex-specific MR effect for the development of anxiety symptoms. This in accordance with studies in which HPA axis functioning and cortisol levels have been consistently related to depression (48–51), but less so to anxiety or only to some forms of anxiety (52). As MR is relevant for the activation and restoration of the HPA axis by binding to cortisol (8, 15), findings for MRs may be comparable to the results of HPA axis functioning and cortisol for anxiety. In line with the findings of Vreeburg et al. (52), anxiety problems are diverse (3), and MR might only relate to the development of certain anxiety symptoms. Moreover, meta-analyses on parenting showed that in general the effects of stressful parenting are smaller for anxiety compared to depression (53, 54), which suggests that the role of MR in the relation between parenting stressors and the development of anxiety symptoms is also smaller. Future research that examines a wide range of internalizing behaviors could shed more light on the extent to which MR CA haplotypes play a role in the development of internalizing behavior problems.

An important strength of our study is the longitudinal investigation of consequences of MR functioning. Whereas most previous research has been cross-sectional and focused on adult populations, we were able to investigate the development across adolescence into adulthood. Moreover, this study was the first that examined MR functioning in relation to other internalizing behavior problems than depression. Despite these strengths, this study also had several limitations. First, we used self-report questionnaires for all constructs. Adolescents’ subjective perceptions of their trauma, the parenting they receive, and their internalizing symptoms are considered the most important information, but using other-reports might have strengthened our findings by providing triangulation. Another disadvantage is that the studied genetic variation was limited to one MR haplotype instead of more extensive genetic variation or HPA axis functionality. However, as earlier studies showed the importance of MRs for stress resilience (8, 9), with a pronounced role for the MR CA haplotype (29), this study on sex-dependent effects of this common and functional MR haplotype was important to gain more insight in why some adolescent are more stress-resilient than others in the development of depressive and anxiety symptoms. Finally, although the sample is quite large for a longitudinal study that covers the developmental period from early adolescence to young adulthood across nine measurement waves, the number of participants might be considered relatively low for analyses including multiple interaction terms. Consequently, we should be cautious with interpretation of our findings given potential low power and relatively few significant associations.

This study showed that MR haplotypes are relevant for the development of depressive symptoms in a community sample, with a protective effect of the MR CA haplotype in girls. No evidence was found for a role of MR in anxiety symptom development, but research on a wider range of internalizing behaviors is needed to clarify the role of MR in the development of internalizing behavior problems. Also, the interaction between MR CA haplotype and environmental stressors warrants further investigation which should take into account biological responses to the timing of the stressor and the amount of stress caused by the stressors. Moreover, in future studies, a more extensive focus on the childhood and early adolescent period, and more details on the role of sex and sex-specific hormones, are needed.

## Data Availability Statement

The datasets generated for this study are available on request to the corresponding author.

## Ethics Statement

The studies involving human participants were reviewed and approved by Medical Ethical Committees of the Utrecht Medical Centre and VU University Medical Centre, Netherlands, and Ethical Committee of Faculty of Social Science of the Utrecht University, Netherlands. Written informed consent to participate in this study was provided by the participants’ legal guardian/next of kin.

## Author Contributions

HE: Formal analysis, writing—original draft, visualization. SN: Formal analysis, writing—review and editing. RS: Formal analysis, writing—review and editing. MB: Conceptualization, writing—review and editing. PL: Conceptualization, writing—review and editing. WM: Conceptualization, writing—review and editing. SB: Conceptualization, writing—review and editing supervision. CV: Conceptualization, writing—original draft, supervision.

## Funding

Data of the RADAR study were used. RADAR has been financially supported by main grants from the Netherlands Organisation for Scientific Research (GB-MAGW 480-03-005, GB-MAGW 480-08-006, GB-MAGW 481-08-014), from a grant to the Consortium Individual Development (Grant 024.001.003) from the Netherlands Organization for Scientific Research, from grants by Stichting Achmea Slachtoffer en Samenleving (SASS), and various other grants from the Netherlands Organisation for Scientific Research, the VU University Amsterdam and Utrecht University.

## Conflict of Interest

The authors declare that the research was conducted in the absence of any commercial or financial relationships that could be construed as a potential conflict of interest.
